# A data-driven model of biomarker changes in sporadic Alzheimer's disease

**DOI:** 10.1093/brain/awu176

**Published:** 2014-07-09

**Authors:** Alexandra L. Young, Neil P. Oxtoby, Pankaj Daga, David M. Cash, Nick C. Fox, Sebastien Ourselin, Jonathan M. Schott, Daniel C. Alexander

**Affiliations:** 1 Centre for Medical Image Computing, Department of Computer Science, University College London, Gower Street, London, WC1E 6BT, UK; 2 Dementia Research Centre, UCL Institute of Neurology, University College London, 8-11 Queen Square, London, WC1N 3AR, UK

**Keywords:** event-based model, disease progression, Alzheimer’s disease, biomarkers, biomarker ordering

## Abstract

We demonstrate the use of a probabilistic generative model to explore the biomarker changes occurring as Alzheimer’s disease develops and progresses. We enhanced the recently introduced event-based model for use with a multi-modal sporadic disease data set. This allows us to determine the sequence in which Alzheimer’s disease biomarkers become abnormal without reliance on *a priori* clinical diagnostic information or explicit biomarker cut points. The model also characterizes the uncertainty in the ordering and provides a natural patient staging system. Two hundred and eighty-five subjects (92 cognitively normal, 129 mild cognitive impairment, 64 Alzheimer’s disease) were selected from the Alzheimer’s Disease Neuroimaging Initiative with measurements of 14 Alzheimer’s disease-related biomarkers including cerebrospinal fluid proteins, regional magnetic resonance imaging brain volume and rates of atrophy measures, and cognitive test scores. We used the event-based model to determine the sequence of biomarker abnormality and its uncertainty in various population subgroups. We used patient stages assigned by the event-based model to discriminate cognitively normal subjects from those with Alzheimer’s disease, and predict conversion from mild cognitive impairment to Alzheimer’s disease and cognitively normal to mild cognitive impairment. The model predicts that cerebrospinal fluid levels become abnormal first, followed by rates of atrophy, then cognitive test scores, and finally regional brain volumes. In amyloid-positive (cerebrospinal fluid amyloid-β_1–42_ < 192 pg/ml) or APOE-positive (one or more APOE4 alleles) subjects, the model predicts with high confidence that the cerebrospinal fluid biomarkers become abnormal in a distinct sequence: amyloid-β_1–42_, phosphorylated tau, total tau. However, in the broader population total tau and phosphorylated tau are found to be earlier cerebrospinal fluid markers than amyloid-β_1–42_, albeit with more uncertainty. The model’s staging system strongly separates cognitively normal and Alzheimer’s disease subjects (maximum classification accuracy of 99%), and predicts conversion from mild cognitive impairment to Alzheimer’s disease (maximum balanced accuracy of 77% over 3 years), and from cognitively normal to mild cognitive impairment (maximum balanced accuracy of 76% over 5 years). By fitting Cox proportional hazards models, we find that baseline model stage is a significant risk factor for conversion from both mild cognitive impairment to Alzheimer’s disease (*P* = 2.06 × 10^−7^) and cognitively normal to mild cognitive impairment (*P* = 0.033). The data-driven model we describe supports hypothetical models of biomarker ordering in amyloid-positive and APOE-positive subjects, but suggests that biomarker ordering in the wider population may diverge from this sequence. The model provides useful disease staging information across the full spectrum of disease progression, from cognitively normal to mild cognitive impairment to Alzheimer’s disease. This approach has broad application across neurodegenerative disease, providing insights into disease biology, as well as staging and prognostication.

## Abstract (precis)

Young *et al.* reformulate an event-based model for the progression of Alzheimer's disease to make it applicable to a heterogeneous sporadic disease population. The enhanced model predicts the ordering of biomarker abnormality in sporadic Alzheimer's disease independently of clinical diagnoses or biomarker cut-points, and shows state-of-the-art diagnostic classification performance.

## Introduction

Existing biomarkers of Alzheimer’s disease provide complimentary information for disease staging and differential diagnosis. Determining the particular sequence and evolution of biomarker abnormality potentially provides a mechanism to stage and stratify patients throughout the full disease time course, and in particular, during the presymptomatic phase. This helps reduce heterogeneity in trial groups, match individuals to putative treatments, and monitor treatment outcomes. Although new diagnostic criteria now incorporate biomarkers to allow earlier diagnosis (Sperling *et al.*, 2011), the evidence base for this is relatively limited. A major challenge of current Alzheimer’s disease research (Jack *et al.*, 2013*a*) is to construct models of disease progression that estimate biomarker ordering and dynamics directly from real-world data sets enabling quantitative evaluation of patient state.

Alzheimer’s disease is characterized pathologically by the build-up of amyloid plaques and neurofibrillary tangles in brain tissue (Braak and Braak, 1991). These pathologies are thought to precede downstream neurodegeneration (i.e. neuronal loss), which leads to clinical symptoms. Biomarkers have been developed that allow the pathological process of Alzheimer’s disease to be monitored *in vivo*. The most well validated of these are CSF amyloid-β_1–42_ (Blennow and Hampel, 2003) and amyloid PET imaging (Klunk *et al.*, 2004; Clark *et al.*, 2011), which measure brain amyloid pathology; CSF phosphorylated tau and total tau (Blennow and Hampel, 2003), as measures of neurofibrillary tangle deposition and neuroaxonal damage; fluorodeoxyglucose (FDG) PET (Herholz, 2012), a measure of brain metabolism; volume and atrophy rate markers derived from structural MRI (Fox and Schott, 2004), which are used to measure the extent and rate of regional neurodegeneration; and cognitive test scores such as the Mini-Mental State Examination (McKhann *et al.*, 1984), which measure cognitive performance.

Hypothetical models of Alzheimer’s disease progression have been proposed (Aisen *et al.*, 2010; Frisoni *et al.*, 2010; Jack *et al.*, 2010) that describe a distinct sequence in which different biomarkers become abnormal. These models generally propose that CSF amyloid-β_1–42_ and amyloid PET abnormalities precede CSF total tau, FDG-PET hypometabolism and atrophy rate measured from structural MRI, which all occur before a clinically significant change in cognitive test scores. However, these models are not informed directly by measured data sets. Jack *et al.* (2011) have attempted to validate the ordering of a subset of these biomarkers: CSF amyloid-β_1–42_, CSF total tau and hippocampal volume; however, their results are dependent on choosing cut points defining abnormal biomarker levels, which are not easy to establish (Bartlett *et al.*, 2012).

Various other attempts to determine biomarker ordering (Lo *et al.*, 2011; Förster *et al.*, 2012; Landau *et al.*, 2012) have used *a priori* staging based on clinical diagnosis. This limits the temporal resolution of these models, typically to three stages (presymptomatic, mild cognitive impairment, and Alzheimer’s disease), and so can provide only a crude ordering of a small number of biomarkers. Other models (Bateman *et al.*, 2012; Buchhave *et al.*, 2012) regress against a particular clinical measure to order biomarkers with better temporal resolution. Bateman *et al.* (2012) use time to disease onset (estimated from subject’s parents for presymptomatic cases) in familial Alzheimer’s disease as the clinical measure. The applicability of these results to sporadic Alzheimer’s disease where the disease may play out differently remains to be determined and depends on accurate estimates of age of symptom onset. A similar approach in sporadic Alzheimer’s disease is to stage subjects retrospectively by time to an Alzheimer’s disease diagnosis. This requires a large elderly cohort to be followed over a long time period to ensure that a significant proportion of the cohort develops Alzheimer’s disease. Buchhave *et al.* (2012) show such an analysis of CSF measures in subjects with mild cognitive impairment. Villemagne *et al.* (2013) instead estimate the rate of change of each biomarker in individuals and integrate over all subjects to get an average biomarker trajectory over time. However, as with the validation provided by Jack *et al.* (2011), cut points are required to determine the ordering of the biomarker trajectories.

The recently introduced event-based model (EBM) (Fonteijn *et al.*, 2012) provides a generative model of disease progression that can learn the ordering of biomarker changes from large cross-sectional (or short-term longitudinal to enable measurement of rates of atrophy) data sets, as well as providing insights into the uncertainty of the reconstructed ordering. The EBM defines the disease progression as a sequence of events at which individual biomarkers become abnormal. The EBM is probabilistic in the sense that it learns normal and abnormal distributions of biomarker values from the data, and so does not require *a priori* staging or cut points. The EBM further enables the assignment of each subject to a disease stage. Previous work (Fonteijn *et al.*, 2012) demonstrated the EBM’s ability to order biomarkers and generate staging measures derived from imaging data, in genetically defined disease and control populations (familial Alzheimer’s disease and Huntington’s disease). However, the original EBM is not directly applicable to sporadic disease data sets, which have significant proportions of misdiagnosed cases in the patient group; and, particularly in Alzheimer’s disease research, a poorly defined control group because a significant number (estimated to be a third by the eighth decade) of apparently healthy elderly individuals have biomarker evidence consistent with presymptomatic Alzheimer’s disease (Rowe *et al.*, 2010; Schott *et al.*, 2010).

Here we reformulate the EBM for multi-modal data from a heterogeneous sporadic disease population. The new EBM accommodates a modest proportion of misdiagnosed patients as well as allowing for presymptomatic cases contaminating the control group. We apply this EBM to the Alzheimer’s Disease Neuroimaging Initiative (ADNI) data set to obtain characteristic biomarker orderings from various subgroups, as well as their uncertainty. We demonstrate the fine-grained staging potential of the EBM and its ability both to classify cognitively normal and Alzheimer’s disease subjects and to predict conversion from mild cognitive impairment to Alzheimer’s disease and cognitively normal to mild cognitive impairment.

## Materials and methods

### Data description

#### Subjects

Data used in the preparation of this article were obtained from the ADNI database (adni.loni.usc.edu). The ADNI was launched in 2003 by the National Institute on Aging (NIA), the National Institute of Biomedical Imaging and Bioengineering (NIBIB), the Food and Drug Administration (FDA), private pharmaceutical companies and non-profit organizations, as a $60 million, 5-year public-private partnership. For up-to-date information, see http://www.adni-info.org. Written consent was obtained from all participants, and the study was approved by the Institutional Review Board at each participating institution.

We downloaded data from LONI (www.loni.ucla.edu/ADNI/) on 5 February 2013, and included all 285 subjects (cognitively normal, mild cognitive impairment or Alzheimer’s disease) that had a CSF examination at baseline, standardized cognitive assessment at baseline (for details see www.adni-info.org/Scientists/Pdfs/adniproceduresmanual12.pdf), which included: the Mini-Mental State Examination (McKhann *et al.*, 1984), the Alzheimer’s Disease Assessment Scale-Cognitive Subscale (ADAS-Cog) (Rosen *et al.*, 1984) (modified 13-item ADAS-Cog, which omits Item 13), and the Rey Auditory Verbal Learning Test (Rey, 1958) (immediate recall score, i.e. the sum of trials 1 to 5), and useable 1.5 T MRI imaging at baseline and 1 year. Clinical diagnosis (cognitively normal/mild cognitive impairment/Alzheimer’s disease) was also recorded. Other possible biomarkers, e.g. FDG-PET and amyloid PET, were not included in the present analysis because they limit the number of available subjects: less than half of subjects with CSF and MRI data at baseline underwent a FDG-PET scan at baseline, and few had baseline amyloid PET imaging. CSF measures of amyloid-β_1–42_, phosphorylated tau and total tau were performed centrally, as previously described (Shaw *et al.*, 2009). The CSF total tau and phosphorylated tau data were log transformed to improve normality. We downloaded *APOE* genotype, for which methods have been published previously (Saykin *et al.*, 2010), for each individual from the LONI website. For validation of the staging system derived from the EBM, we downloaded the aforementioned set of imaging, clinical and CSF data at 12- and 24-month follow-up time points. For the CSF we downloaded longitudinal data over 4 years, so as to obtain baseline, 12- and 24-month CSF data, which were processed in the same batch. As an outcome measure, we downloaded clinical diagnoses at all available time points up to 72 months.

#### Magnetic resonance imaging

Details of the MRI methodology have previously been described (Jack *et al.*, 2008). Cross-sectional regional measures of brain volume for the hippocampus, entorhinal cortex, middle temporal gyrus, fusiform, ventricles and whole brain, as well as total intracranial volume, were calculated at baseline using FreeSurfer Version 4.3, which is documented and freely available for download online (http://surfer.nmr.mgh.harvard.edu/). All regional volumes were normalized by dividing by total intracranial volume for each subject.

Longitudinal measures of regional volume change between 0 and 12 months were obtained using the boundary shift integral (BSI): volume change was measured for the whole brain using the KN-BSI method (Leung *et al.*, 2010*b*), and for the hippocampus using the MAPS-HBSI method (Leung *et al.*, 2010*a*).

#### Event set

The biomarkers available for all the subjects provide the following set of 14 biomarker transition ‘events’, each of which corresponds to a biomarker becoming abnormal, i.e. changing from the ‘control’ to ‘Alzheimer’s disease’ state: (i) three CSF events: amyloid-β_1–42_, phosphorylated tau and total tau; (ii) three cognitive events: ADAS-Cog, Rey Auditory Verbal Learning Test and Mini-Mental State Examination; (iii) six regional brain volume events: brain, ventricles, hippocampus, entorhinal, mid temporal and fusiform volumes; and (iv) two rates of atrophy events: rates of hippocampal and brain atrophy.

### Event sequences

We defined four population subgroups: (i) whole population, all subjects; (ii) amyloid-positive (amyloid+), subjects with CSF amyloid-β_1–42_ < 192 pg/ml. This cut point was chosen according to the results of Shaw *et al.* (2009) who determined cut points using a maximum accuracy classification of autopsy confirmed patients with Alzheimer’s disease and cognitively normal subjects; (iii) APOE-positive (APOE+), subjects with one or more APOE4 alleles; and (iv) amyloid-positive APOE-positive (amyloid+APOE+), subjects who are both amyloid+ and APOE+.

#### The event-based model

We estimated the most likely ordering of events and its uncertainty in each subgroup using the EBM (Fonteijn et al., 2012). The EBM treats each biomarker as either ‘normal’, i.e. non-pathological, or ‘abnormal’, i.e. as seen in Alzheimer’s disease. The switch from normal to abnormal is termed an ‘event’. The occurrence of any particular event, 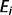, 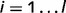, is informed by the corresponding measurements 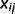 of biomarker 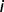 in subject 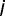, 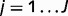. The whole data set 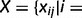 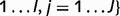 contains measurements of each biomarker in each subject. The most likely ordering of the events is the sequence 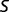 that maximizes the data likelihood
(1)
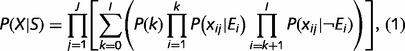

where 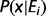 and 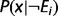 are the likelihoods of measurement 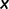 given that biomarker 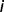 has or has not become abnormal, respectively, and 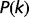 is the prior likelihood of being at stage 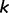, i.e. events 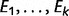 have occurred, and events 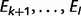 have yet to occur, which we assume is uniform. This uniform prior assumes no knowledge of any patient’s disease stage *a priori*, which imposes the least information possible on estimated orderings.

**Figure 1:**
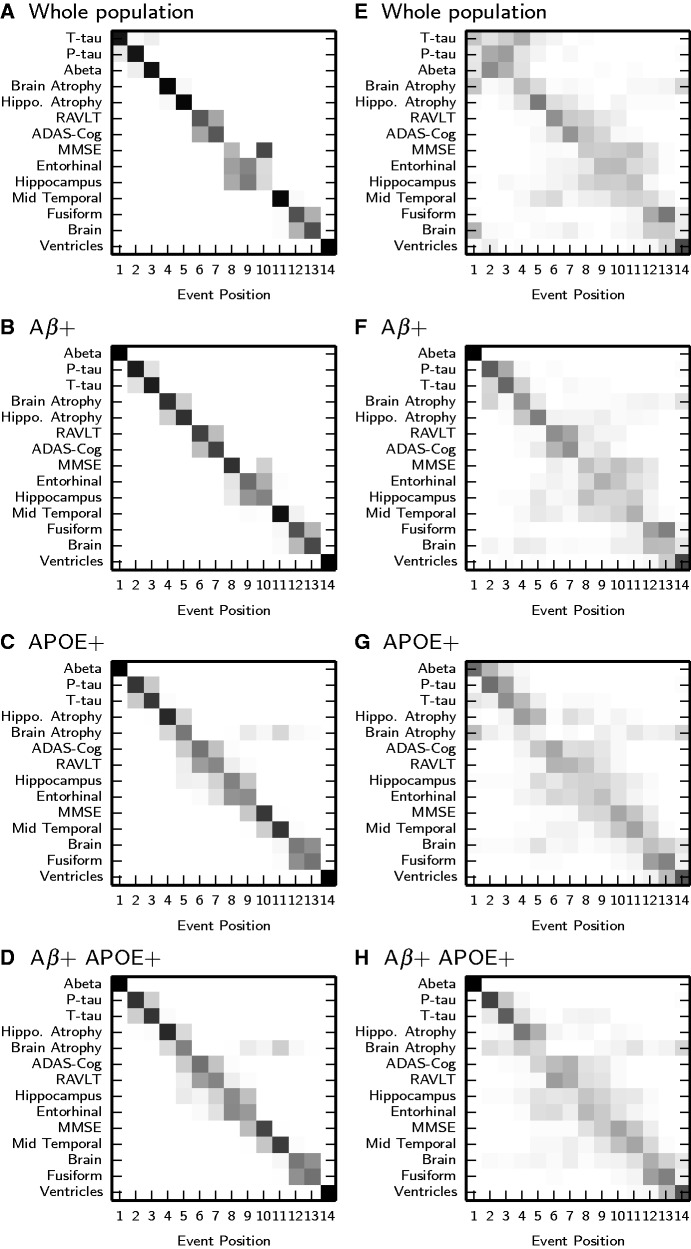
Positional variance diagrams showing the distribution of event sequences in population subgroups. (**A–D**) Positional variance diagrams of the uncertainty in the maximum likelihood event ordering estimated by taking MCMC (Markov chain Monte Carlo) samples using the EBM. (**E–H**) Positional variance diagrams from cross-validation of the maximum likelihood event sequence by bootstrap resampling of the data. These diagrams overestimate the uncertainty, giving a more conservative picture than the left hand column. Each entry in the positional variance diagram represents the proportion of MCMC samples, in **A–D**, or bootstrap samples, in **E–H**, in which events appear at a particular position in the sequence (*x*-axis). This proportion ranges from 0 in white to 1 in black. The *y*-axis orders events by the maximum likelihood sequence. Where rows have a single black block on the diagonal, such as the top five events in the diagram for the whole population, the ordering is strong and permutations of those events are unlikely. Grey blocks, such as the Mini-Mental State Examination (MMSE) score, entorhinal volume and hippocampal volume in the whole population, show that permuting the order of the events has little effect on the likelihood so their ordering is weak. Aβ+ = amyloid+; Abeta = amyloid-β; P-tau = phosphorylated tau; T-tau = total tau; RAVLT = Rey Auditory Verbal Learning Test.

In addition to finding the most likely sequence, we can evaluate 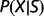 for any sequence to establish the relative likelihood of all sequences. This provides insight into the uncertainty of the ordering. The positional variance diagram (Fonteijn *et al.*, 2012) (Fig. 1A–D) visualizes both the maximum likelihood sequence and its uncertainty by plotting the likelihood that each event appears in each position in the sequence, i.e. the entry of each position is 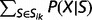 where 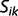 is the set of all sequences with event 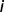 at position 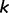.

#### Model of the event distribution

Evaluation of Equation 1 requires models for each of the event distributions, 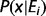 and 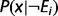. The original EBM in Fonteijn *et al.* (2012) used a familial Alzheimer’s disease data set for which the control group was well defined allowing direct estimation of 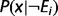. In sporadic Alzheimer’s disease, however, a significant proportion of the cognitively normal control group may have presymptomatic Alzheimer’s disease. To counter this, we fitted a mixture of two normal distributions to each biomarker separately using data from all subjects to obtain the two models. To ensure a robust fit, particularly for biomarkers where the distributions of the healthy and diseased population overlap significantly, we constrain the standard deviations so that the standard deviation of 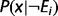 and 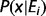 is less than or equal to that of the cognitively normal and Alzheimer’s disease group, respectively. This is a weak constraint designed simply to guide the mixture model away from physically unrealistic solutions. Importantly, while this modelling approach can be used to determine fixed cut points for each biomarker, the model here is not dependent on these cut points, using a probability function to determine the most likely sequencing of event switches.

For specific details of the model fitting procedure for the EBM see the online Supplementary material.

#### Cross-validation of event sequence

We performed cross-validation of the maximum likelihood event sequence returned by the EBM (Fig. 1E–H) by re-estimating the event distributions and maximum likelihood sequence (Supplementary material: Section 1A–B) for 100 bootstrap samples of the data. The positional variance diagrams for the cross validation results show the proportion of bootstrap samples in which event 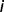 appears at position 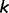 of the maximum likelihood sequence.

### Patient staging

Once the characteristic sequence 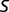 has been determined using the EBM, the simplest way to assign a stage for a particular subject, which we adopt here, is to find the stage which is assigned the highest probability by the model, i.e. the stage,
(2)
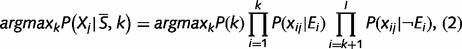

that maximizes the probability of the data given the maximum likelihood event sequence. As before, we make no *a priori* assumptions about model stage by assuming the prior, 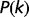, is uniform. The stage ranges from 0 to 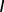 (the number of events). Thus the idealized model for stage *k* is that all events up to and including *k* have occurred and the events after *k* have not occurred. However, the assignment of stage *k* to a particular patient does not mean they fit the model exactly; it is simply the stage most compatible with their measurements.

#### Longitudinal validation

To assess the consistency of patient staging measures longitudinally (Fig. 3) we evaluated each patient’s stage at all follow-up time points that met our inclusion criteria: subjects had to have measurements for all biomarkers, including an MRI scan 12 months later to calculate the boundary shift integral over a consistent time frame. There were two follow-up time points that met these criteria: 12 months (Fig. 3A) and 24 months (Fig. 3B). We compared each subject’s EBM stage at follow-up with their baseline EBM stage, which was re-evaluated using the reprocessed CSF measures so as to ensure that the CSF was processed consistently for all time points.

#### Prediction of conversion

Patient staging derived from the EBM can be used to predict conversion from mild cognitive impairment to Alzheimer’s disease or cognitively normal to mild cognitive impairment (Table 2) by categorizing subjects according to their EBM stage at baseline. We performed a binary classification of mild cognitive impairment subjects into those who have a stable diagnosis of mild cognitive impairment (MCI-stable) and those who convert to Alzheimer's disease (MCI-converters), and cognitively normal subjects into those who have a stable diagnosis of cognitively normal (CN-stable) and those who convert to mild cognitive impairment (CN-converters), by thresholding on patient EBM stage. Stable subjects were defined as those with a mild cognitive impairment or cognitively normal diagnosis who remained with the same diagnosis at the end of a 12-, 24-, 36-, 48- or 60-month follow-up period. Converters were defined as those with a mild cognitive impairment or cognitively normal diagnosis who were diagnosed with Alzheimer’s disease or mild cognitive impairment, respectively, at the end of a 12-, 24-, 36-, 48- or 60-month follow-up period. We used the EBM stage that maximizes balanced accuracy to classify subjects. Balanced accuracy is the average of the sensitivity and specificity, which is similar to accuracy but does not depend on disease prevalence. To test the effect of increasing EBM stage on the probability of conversion from mild cognitive impairment to Alzheimer’s disease and cognitively normal to mild cognitive impairment (Table 3 and Fig. 4), we used Cox proportional hazards models where the event was conversion to Alzheimer’s disease or mild cognitive impairment, respectively and the input variables were patient EBM stage and demographic factors: age, sex, education and *APOE4* carrier status (presence of an *APOE4* allele). Time to event data for subjects who did not convert was considered censored at their last available diagnosis. Statistical significance was set at *P* < 0.05.

### Staging using cross-sectional data alone

To demonstrate the EBM’s ability to stage patients using purely cross-sectional measures we repeated the patient staging by fitting the EBM for a subset of 12 events (Supplementary Tables 1–3 and Supplementary Figs 1–4), excluding atrophy rates. The inclusion criteria were the same as used previously except follow-up MRI scans at 12 months were not required. As before, patient staging results were evaluated for the whole population using the maximum likelihood event sequence determined over all subjects, but with atrophy rates removed (Supplementary Fig. 1A).

## Results

### Subjects

Study subject demographics are summarized in Table 1. Of the 285 subjects that met our inclusion criteria, 189 were amyloid+, 139 were APOE+, and 123 were amyloid+APOE+.

**Table 1:** Baseline demographics for the whole population and population subgroups

	Demographics	Cognitively normal	Mild cognitive impairment	Alzheimer’s disease
All subjects	*n*	92	129	64
	Sex M/F	48/44 (52%)	82/47 (64%)	34/30 (53%)
	Age (years, mean ± SD)	75 ± 5	73 ± 7	75 ± 8
	Education (years, mean ± SD)	15.6 ± 2.9	15.9 ± 3	15 ± 3
	APOE +/−	22/70 (24%)	72/57 (56%)	45/19 (70%)
Amyloid+	*n*	34	96	59
	Sex M/F	19/15 (56%)	58/38 (60%)	31/28 (53%)
	Age (years, mean ± SD)	76 ± 5	73 ± 7	74 ± 8
	Education (years, mean ± SD)	15.8 ± 3.3	15.7 ± 3.1	15 ± 3.1
	APOE +/−	15/19 (44%)	63/33 (66%)	45/14 (76%)
APOE+	*n*	22	72	45
	Sex M/F	15/7 (68%)	39/33 (54%)	25/20 (56%)
	Age (years, mean ± SD)	75 ± 6	73 ± 6	75 ± 7
	Education (years, mean ± SD)	15.6 ± 3.4	15.8 ± 2.9	14.6 ± 3
	APOE +/−	22/0 (100%)	72/0 (100%)	45/0 (100%)
Amyloid+APOE+	*n*	15	63	45
	Sex M/F	10/5 (67%)	35/28 (56%)	25/20 (56%)
	Age (years, mean ± SD)	77 ± 6	73 ± 6	75 ± 7
	Education (years, mean ± SD)	15.5 ± 3.8	15.8 ± 2.9	14.6 ± 3
	APOE +/−	15/0 (100%)	63/0 (100%)	45/0 (100%)

### Event sequences

Figure 1A–D shows positional variance diagrams for each population subgroup. Each positional variance diagram shows the maximum likelihood event sequence and its uncertainty. Figure 1E–H shows positional variance diagrams obtained from cross-validation of the maximum likelihood ordering.

The event sequences in all four populations (Fig. 1A–D) showed broad agreement with hypothetical models such as Jack *et al.* (2010): CSF biomarkers were shown to be early events, followed by atrophy rates, then cognitive test scores and hippocampal and entorhinal volume, and finally other regional brain volumes. Cross-validation (Fig. 1E–H) confirmed high confidence in the ordering of these sets of events: for all populations, the ordering strongly placed CSF and atrophy rates before cognitive test scores and hippocampal and entorhinal volume, and the remaining regional volume changes last.

#### Whole population

The maximum likelihood ordering for the whole population (Fig. 1A) showed some departures from current thinking in neurology (Jack *et al.*, 2010), although the uncertainty was high (Fig. 1E). First, CSF total tau occurred prior to phosphorylated tau. It might be expected that phosphorylated tau is an earlier marker of Alzheimer’s disease than total tau (Jack *et al.*, 2013*a*), being a more specific measure of the build-up of neurofibrillary tangles than total tau (Blennow and Hampel, 2003), which measures associated neuronal damage. Second, both total tau and phosphorylated tau occurred before amyloid-β_1–42_, whereas amyloid plaque deposition is widely considered to be the initiating event in Alzheimer’s disease (Hardy and Selkoe, 2002). Third, brain atrophy rate came before hippocampal atrophy rate, which is at odds with the findings of MRI regional atrophy rate studies (e.g. Scahill *et al.*, 2002).

#### Amyloid+ and APOE+ subjects

The amyloid+, APOE+ and amyloid+APOE+ groups (Fig. 1B–D) showed a distinct ordering of the CSF biomarkers: amyloid-β_1–42_, phosphorylated tau, total tau, which replicated the ordering described by hypothetical models (Jack *et al.*, 2010, 2013*a*). Cross-validation (Fig. 1F–H) of the event sequence in these groups showed a much greater confidence in the ordering of CSF biomarkers compared to the whole population (Fig. 1E), which is more heterogeneous. In the amyloid+ group (Fig. 1B), brain atrophy rate was ordered before hippocampal atrophy rate, but the ordering was weaker than the whole population. In the APOE+ and amyloid+APOE+ groups (Fig. 1C–D) hippocampal atrophy rate clearly occurred before brain atrophy rate.

### Patient staging

#### Cross-sectional distribution of stages

**Figure 2:**
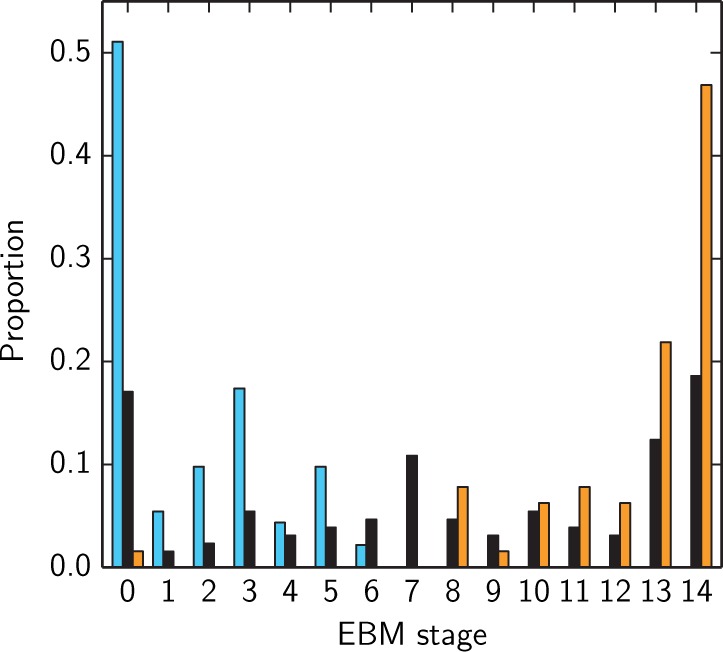
Proportion of patients in each diagnostic category at each EBM stage. Proportion of cognitively normal in light blue, mild cognitive impairment in black, and Alzheimer’s disease in orange. Each EBM stage on the *x*-axis corresponds to the occurrence of a new biomarker transition event. Stage 0 corresponds to no events having occurred and stage 14 is when all events have occurred. Events are ordered by the maximum likelihood event sequence for the whole population as shown in Fig. 1A.

Figure 2 shows the distribution of patient stages for the whole population. All patient staging results were evaluated for the whole population using the maximum likelihood event sequence determined over all subjects (Fig. 1A). The distributions of EBM stages for cognitively normal and Alzheimer’s disease subjects were strongly separated and thresholds at middle stages classify cognitively normal versus Alzheimer’s disease with accuracy >99%. The majority of cognitively normal subjects had no biomarker abnormalities, and were assigned stage 0, or abnormalities only in CSF, and were assigned stages 1–3. A small number of cognitively normal subjects also showed rates of atrophy events, and were assigned stages 4–6. Most subjects with Alzheimer’s disease had abnormal CSF, atrophy rate, cognitive symptoms and low hippocampal and entorhinal volume, and were assigned later stages. The majority of subjects with Alzheimer’s disease were assigned the final stage in the progression, showing that the model configuration that fits their data best is where all of the events have occurred. The distribution of mild cognitive impairment stages overlapped with the distribution of stages for cognitively normal and Alzheimer’s disease subjects, but with a greater concentration of subjects around the middle stages, suggesting that these subjects show CSF abnormalities, abnormal rates of atrophy, and some cognitive symptoms. To explore the extent to which choice of cognitive test affects the staging (and event sequence) output, we assessed the effect of adding in an additional memory test, the Logical Memory II subscale (delayed paragraph recall) from the Wechsler Memory Scale-Revised. Results (not shown) confirm that using this additional cognitive test score provides a similar distribution of patient EBM stages, with logical memory occurring immediately before the Rey Auditory Verbal Learning Test in the event sequence.

#### Longitudinal consistency

**Figure 3:**
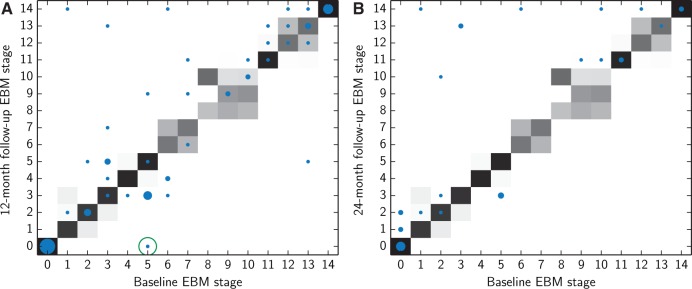
Longitudinal consistency of patient staging in the whole population over a (**A**) 12-month and (**B**) 24-month follow-up period. The size of the dot plotted at each point corresponds to the number of subjects with that particular baseline and follow-up EBM stage. The largest dot, at (0,0) represents 19 subjects in **A** and seven subjects in **B**, and the smallest dots represent one subject. The grey shaded area visualizes the uncertainty in the sequence estimated by the EBM (as shown in Fig. 1A). Subjects whose EBM stage is longitudinally consistent are on or above the line 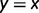 and/or within the grey shaded area. Subjects whose CSF levels (CSF amyloid-β_1–42_ and/or phosphorylated tau and/or total tau) change from an abnormal to a normal level at follow-up are circled in green.

Figure 3 compares each subject’s EBM stage at baseline with their EBM stage at 12- and 24-month follow-ups. Patient staging showed good longitudinal consistency, with the EBM stage of each subject generally increasing or remaining stable at each follow-up (most points are within or above the grey shaded area, which represents the uncertainty estimated by the EBM, as shown in Fig. 1A). The small number of individuals whose EBM stage decreased longitudinally (below the diagonal) by more than the uncertainty estimated by the EBM (shaded in grey) were all subjects who improved from an abnormal to a normal score on one or more of the three cognitive tests (Mini-Mental State Examination, Rey Auditory Verbal Learning Test, and ADAS-Cog) and/or two atrophy rates (brain atrophy rate and hippocampal atrophy rate) with the exception of one subject (circled in green) whose CSF amyloid-β_1–42_ levels increased from a clearly abnormal level of 139 pg/ml at baseline to a more borderline level of 207 pg/ml at the 12-month follow-up.

#### Prediction of clinical outcomes

Table 2 shows the balanced accuracy, sensitivity, specificity, area under the receiver operating characteristic (ROC) curve, and maximum accuracy threshold EBM stage for classification of MCI-stable versus MCI-converters over different follow-up durations. The balanced accuracy and area under the ROC curve of the classification were comparable to state-of-the-art classification techniques (Young *et al.*, 2013). As the duration of the follow-up increased, the maximum balanced accuracy threshold decreased, i.e. later EBM stages were better at predicting faster conversion times. These optimal stage thresholds suggest that abnormal CSF measures, atrophy rate, cognitive test scores and hippocampal and entorhinal volume provide the best prediction of conversion in ≤2 years, whereas just abnormal CSF, atrophy rate and ADAS-Cog and Rey Auditory Verbal Learning Test scores is the combination that best predicts conversion over a period of 3 to 5 years.

**Table 2:** Classification results for discriminating MCI-stable versus MCI-converters and CN-stable versus CN-converters using patient stage at baseline

	Balanced accuracy (%)	Sensitivity (%)	Specificity (%)	AUC	Threshold stage	*n*-c/*n*-s
**MCI-converters versus MCI-stable**						
12 months	67	60	73	0.69	12	30/96
24 months	68	57	80	0.71	12	53/64
36 months	77	86	69	0.78	7	65/48
48 months	78	83	72	0.76	7	70/18
60 months	76	84	69	0.77	7	73/16
**CN-converters versus CN-stable**						
12 months	84	100	68	0.76	3	2/90
24 months	66	67	66	0.62	3	6/83
36 months	68	63	73	0.62	3	8/73
48 months	66	58	74	0.65	3	12/49
60 months	76	75	76	0.75	2	16/38

Threshold stage is the maximum balanced accuracy EBM stage for separating stable subjects from converters. Subjects with a baseline EBM stage less than this threshold are classified as stable and subjects with a baseline EBM stage greater than or equal to this threshold are classified as converters.

AUC = area under receiver operating characteristic curve; *n*-c = number of converters, *n*-s = number of stable subjects.

The same statistics are shown in Table 2 for classification of CN-stable versus CN-converters. Again the threshold EBM stage decreased for increasing follow-up durations, with abnormal CSF total tau, phosphorylated tau and amyloid-β_1–42_ levels best predicting conversion from cognitively normal to mild cognitive impairment over a period of ≤4 years, but just abnormal CSF total tau and phosphorylated tau best predicting conversion over 5 years.

**Figure 4:**
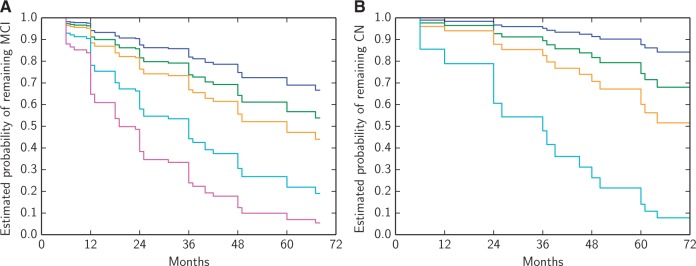
Estimated probability of remaining (**A**) mild cognitive impairment or (**B**) cognitively normal for different baseline EBM stages, obtained by fitting Cox proportional hazards models. These estimated probabilities are shown for the average population demographics (74.1 years of age, 15.6 years of education, APOE−, male sex). Stages are grouped so that normal (blue) = stage 0, CSF (green) = stages 1–3, atrophy (orange) = stages 4–5, cognition (cyan) = stages 6–10, which includes hippocampal and entorhinal volume as well as cognitive test scores, volume (magenta) = stages 11–14. See Supplementary Fig. 5 for an extended version of this figure, which includes a table of the number of subjects at risk at each follow-up time point. MCI = mild cognitive impairment; CN = cognitively normal.

Table 3 shows the hazard ratio and statistical significance of each variable in the Cox proportional hazards models. Increasing EBM stage was a significant hazard for conversion from both mild cognitive impairment to Alzheimer’s disease and cognitively normal to mild cognitive impairment. Figure 4 shows the estimated probability of remaining cognitively normal or mild cognitive impairment depending on baseline EBM stage.

**Table 3:** Hazard ratios with 95% confidence intervals (CI) for conversion from mild cognitive impairment to Alzheimer’s disease, and cognitively normal to mild cognitive impairment, obtained by fitting uncorrected and corrected Cox proportional hazards models

	Hazard ratio (CI)	*P*-value	Corrected hazard ratio (CI)	Corrected *P*-value
**MCI to Alzheimer’s disease progression**				
EBM stage	1.15 (1.09–1.21)	1.58 × 10^−7^*	1.15 (1.09–1.21)	2.06 × 10^−7^*
Age	0.99 (0.96–1.03)	0.68	0.99 (0.96–1.02)	0.49
Education	0.98 (0.91–1.05)	0.55	0.98 (0.90–1.05)	0.51
APOE4 carrier	1.55 (0.97–2.48)	0.065	1.19 (0.73–1.94)	0.49
Male	0.77 (0.49–1.23)	0.28	0.85 (0.50–1.45)	0.55
**Cognitively normal to MCI progression**				
EBM stage	1.34 (1.07–1.69)	0.012*	1.31 (1.02–1.68)	0.033*
Age	0.99 (0.90–1.09)	0.84	0.98 (0.89–1.08)	0.67
Education	1.03 (0.88–1.22)	0.69	1.02 (0.86–1.20)	0.83
APOE4 carrier	3.15 (1.19–8.30)	0.021*	2.47 (0.85–7.17)	0.096
Male	1.75 (0.65–4.74)	0.27	1.45 (0.49–4.28)	0.5

*P < 0.05. MCI = mild cognitive impairment.

### Staging using cross-sectional data alone

We repeated all analyses for purely cross-sectional measures, i.e. excluding rates of atrophy, to demonstrate the clinical application of our staging system, where patients need to be staged at one point in time. Supplementary Table 1 gives demographic information for the 325 subjects that met our inclusion criteria, of which 216 were amyloid+, 159 were APOE+, and 141 were amyloid+APOE+.

Removing atrophy rates had little effect on biomarker ordering (Supplementary Fig. 1) or the cross-sectional distribution (Supplementary Fig. 2) and longitudinal consistency (Supplementary Fig. 3) of staging. Again, individuals whose EBM stage decreased longitudinally (below the diagonal) by more than the uncertainty estimated by the EBM (shaded in grey) improved from a clearly abnormal to a more normal score on one or more of the three cognitive tests (Mini-Mental State Examination, Rey Auditory Verbal Learning Test, and ADAS-Cog) with the exception of two subjects (circled in green) whose CSF levels (CSF amyloid-β_1–42_ and/or phosphorylated tau and/or total tau) changed from an abnormal to a more normal level at follow-up.

The balanced accuracy for predicting conversion (Supplementary Table 2) was slightly reduced when the atrophy rates were removed but was still high, giving a maximum balanced accuracy of 71% (77% with atrophy rates) for conversion from mild cognitive impairment to Alzheimer’s disease over 3 years, and 70% (76% with atrophy rates) for conversion from cognitively normal to mild cognitive impairment over 5 years. On average over all follow-up durations, the balanced accuracy decreased by 2.6% for predicting conversion from mild cognitive impairment to Alzheimer’s disease, and increased by 4% for predicting conversion from cognitively normal to mild cognitive impairment. Again, increasing EBM stage was a significant hazard for conversion from both mild cognitive impairment to Alzheimer’s disease and cognitively normal to mild cognitive impairment (Supplementary Table 3 and Supplementary Fig. 4).

## Discussion

We have adapted the EBM for use with multi-modal sporadic disease data sets to determine the characteristic ordering of biomarker transitions and provide a staging system for disease monitoring. We use the EBM here to derive characteristic biomarker orderings in Alzheimer’s disease from various subgroups of the ADNI data set and to provide insight into the variability of the ordering. The orderings provide detailed information on the dynamics of large sets of biomarkers across the full duration of Alzheimer’s disease progression. They describe a distinct sequence of biomarker transitions in which CSF measures are the earliest to become abnormal, followed by atrophy rates, and finally cognitive test scores and regional brain volumes. The recovered ordering shows less variation in the sequence for amyloid+, APOE+ or amyloid+APOE+ individuals than for the whole population, most likely reflecting that the former are a more homogeneous group with archetypical Alzheimer’s disease pathology. The results of the EBM provide entirely data-driven support for hypothetical models of Alzheimer’s disease progression, such as Aisen *et al.* (2010), Frisoni *et al.* (2010) and Jack *et al.* (2010), without the requirement for determining biomarker cut-points (Bartlett *et al.*, 2012).

The staging system provides a much more detailed evaluation of patient state than clinical diagnoses. Importantly, it has clear clinical relevance, providing a high accuracy classification of cognitively normal versus Alzheimer’s disease subjects, predicting conversion from mild cognitive impairment to Alzheimer’s disease and cognitively normal to mild cognitive impairment, and being applicable not only to short-term longitudinal data sets (allowing atrophy measurements), but also to fully cross-sectional data sets (one visit).

### Event sequence

#### Ordering of cerebrospinal fluid biomarkers

The ordering of the CSF biomarkers in amyloid+ and APOE+ individuals supports the ordering of CSF biomarkers predicted by earlier hypothetical models of Alzheimer’s disease progression: CSF amyloid-β_1–42_, phosphorylated tau, total tau. Because amyloid+ individuals are likely to have early Alzheimer’s disease, this group should represent a much purer Alzheimer’s disease population than the whole population and thus the biomarker ordering should reflect the Alzheimer’s disease ordering more closely. Similarly, *APOE4* carriers would also be predicted to shown this pattern, given the very strong association between *APOE4* and amyloid-β deposition (Andreasson *et al.*, 2013).

In the broader population, however, our results suggest that CSF total tau and phosphorylated tau may become abnormal before amyloid-β_1–42_, i.e. that there are a significant proportion of subjects who have CSF total tau and phosphorylated tau, but not amyloid-β_1–42_ abnormalities, although cross-validation shows higher uncertainty. Given the results in the APOE+ and amyloid+ populations, it seems likely that these subjects reside predominantly in the APOE− and amyloid− populations, and indeed estimation of the ordering using the APOE− and amyloid− subject groups alone supports this hypothesis, confirming that CSF total tau and phosphorylated tau events appear earlier than CSF amyloid-β_1–42_ (data not shown). As discussed recently (Jack *et al.*, 2013*a*; Jack and Holtzman, 2013*b*), there are several potential explanations for this finding. First, that tau accumulation is a common feature of aging. Braak and Del Tredici (2011) found tau pathology to be present in healthy individuals at autopsy from as early as 20 years of age. These findings are replicated by the study of Kok *et al.* (2009), which found neurofibrillary tangle deposition in a significant proportion of APOE− individuals between 30 and 59 years of age. Our results, which demonstrate discrepancies between the ordering in APOE+ and APOE− individuals, would be entirely consistent with these findings, with the pattern observed in the population as a whole reflecting a mixture of two populations: one already on the path to developing Alzheimer’s disease, the other undergoing normal aging, with total tau and phosphorylated tau a common early feature in both. A second alternative is that accumulation of tau pathology may be an early feature of Alzheimer’s disease either for some or all subjects. Early tau pathology may be more prevalent in APOE− and amyloid− individuals, or alternatively, as the subjects recruited for ADNI are age-matched, we might not observe early tau pathology in the APOE+ and amyloid+ populations who would be likely to develop Alzheimer’s disease at a younger age, and thus already have abnormal amyloid levels. A third possibility is that amyloid accumulation does precede tau deposition, but that either current CSF amyloid-β_1–42_ assays are less sensitive than the CSF total tau and phosphorylated tau assays, or do not detect the earliest (e.g. oligomeric) abnormal amyloid-β moieties. Finally, as CSF total tau is not specific to Alzheimer’s disease and is found in other neurodegenerative diseases, e.g. stroke, trauma and encephalitis (Blennow *et al.*, 2010), a further alternative is that individuals have other, perhaps presymptomatic neurodegenerative diseases, such as frontotemporal dementa, or dementia with Lewy bodies. Such individuals might be under-represented in the APOE+ and/or amyloid+ groups, which are enriched for Alzheimer’s disease, and thus more prevalent in the APOE− and amyloid− groups.

#### Ordering of magnetic resonance imaging biomarkers

The ordering of MRI biomarkers from the EBM agrees with previous findings (Thompson *et al.*, 2001; Scahill *et al.*, 2002), with atrophy rates becoming abnormal before overall volume changes, and volume changes occurring in a distinct sequence, starting in the hippocampus and entorhinal cortex, progressing to other temporal lobe areas, the middle temporal gyrus and the fusiform gyrus, with resulting overall brain volume loss and ventricular expansion. Results in APOE+ subjects also support previous findings (Schuff *et al.*, 2009; Caroli and Frisoni, 2010), suggesting earlier hippocampal and entorhinal volume loss, which occur before Mini-Mental State Examination reduction in the APOE+ population and after Mini-Mental State Examination in the whole population and amyloid+ population.

One perhaps surprising result of the MRI biomarker ordering is that the increasing whole brain atrophy rate event occurs before the hippocampal atrophy rate event both in the whole and amyloid-β+ population. In common with any data-driven model of biomarker changes, the EBM orders events based on when the corresponding measurements become discernibly different between cases and controls. This may not reflect the order of appearance of underlying pathology as the precision of the measurements may vary (Fonteijn *et al.*, 2012). Thus, this result might simply reflect the increased variability associated with measurement of hippocampal over whole-brain atrophy rates (Leung e*t al*., 2013). Other possible factors are that the results are influenced by subjects who have a mixture of pathologies, where other processes occur alongside Alzheimer’s disease that contribute to brain atrophy rate but not hippocampal atrophy rate, such as vascular disease (Barnes *et al.*, 2013), or other neurodegenerative diseases (Whitwell *et al.*, 2007). Alternatively, excess whole brain atrophy may be a core feature of all patients with Alzheimer’s disease, noting that some individuals with pathologically confirmed Alzheimer’s disease have relatively hippocampal sparing disease (Whitwell *et al.*, 2012).

#### Uncertainty in the event sequence

The uncertainty in the event sequence, as shown by the positional variance diagrams and cross-validation results, potentially provides useful information about the variation of biomarker ordering across the population. However, three main factors contribute to the uncertainty. First, natural variation: some events may occur in different orders in different individuals. For example, for APOE+ subjects, hippocampal volume loss may occur earlier than in APOE− subjects (Schuff *et al.*, 2009; Caroli and Frisoni, 2010); thus in the whole population that combines both groups, uncertainty is higher. Second, sampling density: when events occur in close succession, there are likely to be fewer of the data points, which are required to determine their ordering, that separate them. Third, outliers: the data set may include subjects who do not follow any typical progression pattern of Alzheimer’s disease, e.g. subjects with other neurodegenerative diseases. Although the model fitting procedure we use is somewhat robust to these outliers, they can still affect the posterior distribution on the ordering, which manifests as uncertainty.

#### Using the event-based model to define cut points

A major advantage of the EBM is that the ordering of biomarkers is not dependent on cut points. Instead, the EBM is probabilistic, calculating the probability that each event has occurred from models of the distributions of normal and abnormal biomarkers learned from the data rather than assuming an event has occurred when a certain threshold value is reached. However, for comparison we derived cut point values, given in Table 4, which represent the point at which the biomarker value is equally likely to be normal or abnormal, and should therefore be similar to existing biomarker cut points. The resulting cut points for the CSF biomarkers are similar to those reported by Shaw *et al.* (2009), which were derived using a maximum accuracy classification of autopsy confirmed Alzheimer’s disease versus healthy controls. Importantly, the ordering provided by the EBM can be seen not merely to reflect the ordering of the sensitivity or specificity of these cut points.

**Table 4:** Cut point values derived using the event distributions estimated by the EBM

Biomarker	Cut point	Sensitivity (%)	Specificity (%)
Amyloid-β (pg/ml)	189	92	63
Phosphorylated tau (pg/ml)	25	88	71
Total tau (pg/ml)	80	77	73
Hippocampal atrophy (ml/year)	0.138	72	75
Brain atrophy (ml/year)	11.9	64	78
RAVLT	33	92	91
ADAS-Cog	17	97	97
MMSE	27	100	97
Hippocampus (% TIV)	0.423	81	82
Entorhinal (% TIV)	0.214	84	83
Mid temporal (% TIV)	1.19	75	78
Whole brain (% TIV)	64.6	73	66
Fusiform (% TIV)	1.05	73	73
Ventricles (% TIV)	3.04	48	85

Volume measurements (hippocampus, entorhinal, mid temporal, fusiform, whole brain, ventricles) are summed over the left and right hemisphere and total intracranial volume normalized, and are recorded as a percentage of the total intracranial volume. The sensitivity is the percentage of Alzheimer’s disease subjects with abnormal measurements, and specificity is the percentage of cognitively normal subjects with normal measurements, when subjects are classified using these cut points.

MMSE = Mini-Mental State Examination; RAVLT = Rey Auditory Verbal Learning Test; TIV = total intracranial volume.

### Patient staging

A more directly practical output of the EBM is the data-driven staging system it provides. Here we demonstrate, for the first time, the use of such a patient staging measure to predict clinical outcomes. Our staging measure strongly separates cognitively normal and Alzheimer’s disease subjects and gives comparable results to state-of-the-art classification techniques for prediction of conversion from mild cognitive impairment to Alzheimer’s disease (Young *et al.*, 2013), albeit with a larger set of biomarkers. The major advantage of the EBM, a generative model, is that it explicitly provides useful information on what drives the classification, unlike the discriminative models used in Young *et al.* (2013). We used the EBM’s staging system to predict conversion from cognitively normal to mild cognitive impairment, as well as mild cognitive impairment to Alzheimer’s disease, and over different follow-up durations. The classification results are supported by the results of the Cox proportional hazards models, which find EBM stage to be a significant hazard for conversion from both mild cognitive impairment to Alzheimer’s disease and cognitively normal to mild cognitive impairment. This suggests that the EBM, once sufficient control/Alzheimer’s disease data are available, might have clinical application, providing valuable prognostic information on an individual patient basis, and potentially for clinical trial stratification.

### Model assumptions

When interpreting these results, it is important to stress that the EBM is based on strong assumptions, which are explicitly designed to simplify reality to determine major trends in data. This section summarizes the key assumptions made in the modelling process, their potential influence on results, and possibilities to relax the assumptions in future work.

#### Event sequence

The EBM, like other data-driven models (Jack *et al.*, 2011; Lo *et al.*, 2011; Bateman *et al.*, 2012; Buchhave *et al.*, 2012; Förster *et al.*, 2012; Landau *et al.*, 2012; Villemagne *et al.*, 2013), assumes that all subjects follow a single progression pattern. While this may be reasonable for the amyloid+ and APOE+ groups, the wider sporadic Alzheimer’s disease is likely to show more variability in the event sequence due to the inherent disease heterogeneity, driven perhaps by genetic, e.g. the presence or absence of APOE4 (Schott *et al.*, 2006), or lifestyle factors. The single sequence the EBM identifies maximizes compatibility within the set of subjects. It is thus important to consider not only the most likely sequence, but also the positional variance diagram and cross-validation output, which explicitly highlight areas of uncertainty, aiding interpretation particularly where the data depart from the assumptions, for example in heterogeneous groups. The positional variance diagrams generated directly from the EBM (Fig. 1A–D) underestimate the uncertainty in the event ordering, as they do not account for uncertainty in the biomarker distribution models. The cross-validation results (Fig. 1E–H), on the other hand, tend to overestimate the uncertainty, because each iteration considers only a subset of the data. In our whole-population analysis, both mechanisms show reasonable stability of the results, which gives some confidence to the conclusions. However, it is important to remember that the single sequence does not represent all subjects and the positional variance diagrams are only a crude indicator of heterogeneity of the event sequence. More sophisticated models that can relax the assumption of a single event ordering, (e.g. Beckett, 1993; Huang and Alexander, 2012), and/or provide uncertainty estimates by modelling the uncertainty in the biomarker distribution parameters, are important areas for future study.

#### Patient staging

Although the modelling approach provides a powerful potential means of patient staging, it is important that such staging information is interpreted correctly. While the idealized model for, e.g. stage 3, is that all CSF biomarkers are abnormal and all others are normal, a patient assigned stage 3 need not fit this profile exactly; stage 3 is simply the idealized stage most compatible with a given individual’s biomarker measurements. This formulation enables the EBM to stage subjects who do not conform to the maximum likelihood event sequence, which is important given the heterogeneity of sporadic Alzheimer’s disease. Despite its idealized nature, the staging system has clear clinical relevance, as demonstrated by the strong classification performance and Cox proportional hazards model results; those results also add confidence to the event sequence derived from the whole population, which underpins the staging. The probabilistic nature of the staging system presents opportunities for refinement in future work. Here we assign only the most likely stage, but using Equation 2 we can quantify the uncertainty in the stage assignment, which may contain useful additional diagnostic and prognostic information. Moreover, also using Equation 2, we can obtain an overall likelihood of conforming to the event sequence, which should be useful for detecting misdiagnoses or choosing the most likely diagnosis from a selection of models for different diseases.

### Further applications

The EBM offers a range of possibilities for wider application. This work focuses on regional imaging measures, CSF and cognitive biomarkers. Future work will determine the ordering of other Alzheimer’s disease biomarkers and a more extensive set of regional imaging biomarkers as in Fonteijn *et al.* (2012). In particular, including FDG and amyloid PET biomarkers, which may help separate mild cognitive impairment subtypes (Prestia *et al.*, 2013), and in due course tau-PET will be of considerable interest. This may also be possible by refining the EBM to allow for missing data, which would enable it to recover ordering from incomplete data sets; this would also enable reliable models of the amyloid– and APOE− groups. Currently the ADNI data set is the only freely available data set that has a sufficiently large number of subjects, and diversity of biomarkers to support the EBM analysis. Repetition of these analyses on other Alzheimer’s disease data sets will provide important validation of our results. An EBM formulation that allows for missing data could use a range of data sets as input, and output combined results. Work on such a formulation requires careful statistical evaluation and is on-going. Application of the EBM to other dementias, such as the various forms of (sporadic and familial) frontotemporal dementia, vascular dementia or dementia with Lewy bodies, will provide insight into how the underlying pathological process varies across different types of dementia. It offers the possibility to obtain staging systems for other diseases, as we show here for Alzheimer’s disease. Moreover, the generative nature of the EBM enables differential diagnosis, as the EBM can assign a likelihood of a particular case fitting the sequence for any particular disease. Furthermore, the technique can be applied to any sequential mechanism, and so naturally extends to model a wide variety of other diseases or developmental processes (such as skill acquisition or normal aging).

## Conclusion

We have developed a data-driven model for determining biomarker ordering and staging patients. We have used the model with the ADNI data set to support currently hypothetical models, but further to highlight uncertainty in those orderings and variation among different subgroups. We also demonstrate that such a model can provide a practical and effective staging system for patient prognosis.

## Supplementary Material

Supplementary Data

## Abbreviations

ADAS-Cog: Alzheimer’s Disease Assessment Scale-Cognitive Subscale
ADNI: Alzheimer’s Disease Neuroimaging Initiative
CN-converters: cognitively normal subjects who convert to mild cognitive impairment at follow-up
CN-stable: cognitively normal subjects with a stable cognitively normal diagnosis at follow-up
EBM: event-based model
FDG: fluorodeoxyglucose
MCI-converters: mild cognitive impairment subjects who convert to Alzheimer’s disease at follow-up
MCI-stable: mild cognitive impairment subjects with a stable mild cognitive impairment diagnosis at follow-up
