# Supplementary material for: A data-driven model of biomarker changes in sporadic Alzheimer's disease
Source: Brain. 2014 Jul 9;137(9):2564–77. doi: 10.1093/brain/awu176 (PMC4132648; doi:10.1093/brain/awu176)
Supplement: Supplementary Data [file supp_137_9_2564__index.html]

A data-driven model of biomarker changes in sporadic Alzheimer's disease — A data-driven model of biomarker changes in sporadic Alzheimer's disease — Supplementary Data 

# A data-driven model of biomarker changes in sporadic Alzheimer's disease

## Supplementary Data

files

**Files in this Data Supplement:**

- Supplementary Data - docx file
