# Supplementary material for: A data-driven model of biomarker changes in sporadic Alzheimer's disease
Source: Brain. 2014 Jul 9;137(9):2564–77. doi: 10.1093/brain/awu176 (PMC4132648; doi:10.1093/brain/awu176)
Supplement: Supplementary Data [file supp_awu176_SupplementaryMaterial_Revised_V2.docx]

**Supplementary Methods**

**Section S1: Summary of Estimation Procedure for EBM**

1. Fit a mixture model to the data for all subjects to estimate the parameters of the event distributions, $P(x|E_{i})$ and $P(x|\neg E_{i})$

For each population subgroup:

1. Find the characteristic event sequence $\bar{S}$ which maximizes the data likelihood $P(X|S)$ by performing a greedy ascent algorithm
2. Take MCMC samples of the data likelihood $P(X|S)$, initialized from the maximum likelihood event sequence $\bar{S}$, to estimate the uncertainty in the characteristic event sequence.

**Additional Experiments**

To demonstrate the clinical application of our staging system, where patients need to be staged at one point in time, we repeated all experiments using purely cross-sectional measures, i.e. excluding rates of atrophy. The results are shown in Tables S1-S3 and Figures S1-S4.

|  | Demographics | Cognitively normal | Mild cognitive impairment | Alzheimer’s disease |
| --- | --- | --- | --- | --- |
| All subjects | N | 100 | 150 | 75 |
|  | Sex M/F | 51/49 (51%) | 98/52 (65%) | 41/34 (55%) |
|  | Age (years, mean ± SD) | 75±5 | 73±7 | 75±8 |
|  | Education (years, mean ± SD) | 15.7±2.9 | 15.7±3 | 15.1±3 |
|  | APOE +/- | 22/78 (22%) | 83/65 (57%) | 52/23 (69%) |
| Amyloid+ | N | 36 | 111 | 69 |
|  | Sex M/F | 20/16 (56%) | 69/42 (62%) | 38/31 (55%) |
|  | Age (years, mean ± SD) | 76±5 | 74±7 | 74±8 |
|  | Education (years, mean ± SD) | 15.9±3.3 | 15.6±3.1 | 15±3 |
|  | APOE +/- | 15/21 (42%) | 74/37 (67%) | 52/17 (75%) |
| APOE+ | N | 22 | 85 | 52 |
|  | Sex M/F | 15/7 (68%) | 49/36 (58%) | 30/22 (58%) |
|  | Age (years, mean ± SD) | 75±6 | 73±6 | 74±8 |
|  | Education (years, mean ± SD) | 15.6±3.4 | 15.6±3 | 14.8±3 |
|  | APOE +/- | 22/0 (100%) | 85/0 (100%) | 52/0 (100%) |
| Amyloid+ APOE+ | N | 15 | 74 | 52 |
|  | Sex M/F | 10/5 (67%) | 43/31 (58%) | 30/22 (58%) |
|  | Age (years, mean ± SD) | 77±6 | 74±7 | 74±8 |
|  | Education (years, mean ± SD) | 15.5±3.8 | 15.7±2.9 | 14.8±3 |
|  | APOE +/- | 15/0 (100%) | 74/0 (100%) | 52/0 (100%) |

**Table S1**

Baseline demographics for whole population and population subgroups when atrophy rates are removed.

**Figure S1**

As Figure 1, but without using atrophy rates, i.e. using the subjects in Table S1.

**Figure S2**

As Figure 2, but without using atrophy rates. Events are ordered by the maximum likelihood event sequence for the whole population as shown in Figure S1A.

**Figure S3**

As Figure 3, but without using atrophy rates. Two additional follow up time points, at 36 and 48 months, met our inclusion criteria. Here, the largest dot, at (0,0) represents 17 subjects in (A) and 9 subjects in (B). The largest dot in (C) is at (12,12) and represents 2 subjects. In (D) all dots represent 1 subject.

| **A. MCI-converters vs. MCI-stable** | | |  |  |  |  |
| --- | --- | --- | --- | --- | --- | --- |
|  | Balanced accuracy (%) | Sensitivity (%) | Specificity (%) | AUC | Threshold stage | N-c/N-s |
| 12 months | 67 | 72 | 62 | 0.71 | 8 | 32/103 |
| 24 months | 68 | 68 | 68 | 0.71 | 8 | 57/68 |
| 36 months | 71 | 83 | 59 | 0.74 | 6 | 69/51 |
| 48 months | 74 | 84 | 65 | 0.71 | 5 | 74/20 |
| 60 months | 73 | 84 | 63 | 0.74 | 5 | 77/16 |
|  |  |  |  |  |  |  |
| **B. CN-converters vs. CN-stable** | | |  |  |  |  |
|  | Balanced accuracy (%) | Sensitivity (%) | Specificity (%) | AUC | Threshold stage | N-c/N-s |
| 12 months | 95 | 100 | 91 | 0.95 | 4 | 2/95 |
| 24 months | 79 | 67 | 91 | 0.78 | 4 | 6/86 |
| 36 months | 70 | 45 | 95 | 0.69 | 4 | 9/76 |
| 48 months | 66 | 38 | 94 | 0.68 | 4 | 13/50 |
| 60 months | 70 | 76 | 64 | 0.75 | 1 | 17/39 |

**Table S2**

As Table 2, but without using atrophy rates.

| **A. MCI to Alzheimer’s disease progression** | | | | | |  |  | | |  | | |
| --- | --- | --- | --- | --- | --- | --- | --- | --- | --- | --- | --- | --- |
|  | Hazard ratio (CI) | *P*-value | | Corrected hazard ratio (CI) | | | | Corrected *P*-value | | |  |  |
| EBM Stage | 1.16 (1.10-1.23) | 3.34 x 10^-7^* | | 1.17 (1.10-1.24) | | | | 3.55 x 10^-7^* | | |  |  |
| Age | 1.00 (0.97-1.03) | 0.98 | | 0.99 (0.96-1.02) | | | | 0.51 | | |  |  |
| Education | 0.98 (0.91-1.06) | 0.65 | | 0.98 (0.91-1.06) | | | | 0.6 | | |  |  |
| APOE4 Carrier | 1.56 (0.98-2.46) | 0.059 | | 1.32 (0.82-2.13) | | | | 0.25 | | |  |  |
| Male | 0.78 (0.49-1.22) | 0.27 | | 0.84 (0.50-1.43) | | | | 0.52 | | |  |  |
|  |  |  | |  | | | |  | | |  |  |
| **B. Cognitively normal to MCI progression** | | |  | |  | | | |  | | |  |
|  | Hazard ratio (CI) | *P*-value | | Corrected hazard ratio (CI) | | | | Corrected *P*-value | | |  |  |
| EBM Stage | 1.66 (1.29-2.14) | 1.01 x 10^-4^* | | 1.59 (1.22-2.09) | | | | 6.72 x 10^-4^* | | |  |  |
| Age | 1.00 (0.91-1.10) | 0.99 | | 0.99 (0.90-1.09) | | | | 0.83 | | |  |  |
| Education | 1.02 (0.88-1.21) | 0.76 | | 0.99 (0.84-1.15) | | | | 0.85 | | |  |  |
| APOE4 Carrier | 3.00 (1.16-7.78) | 0.024* | | 2.02 (0.68-6.00) | | | | 0.21 | | |  |  |
| Male | 2.00 (0.75-5.33) | 0.17 | | 1.38 (0.46-4.14) | | | | 0.57 | | |  |  |

**Table S3**

As Table 3, but without using atrophy rates.

**Figure S4**

As Figure 4, but without using atrophy rates. These estimated probabilities are shown for the average population demographics (74.2 years of age, 15.6 years of education, APOE negative, male sex). Stages are grouped analogously to Figure 4, so that here Normal (blue) = stage 0, CSF (green) = stages 1-3, Cognition (cyan) = stages 4-8, which includes hippocampal and entorhinal cortex volume as well as cognitive test scores, Volume (magenta) = stages 9-12.

**Figure S5**

As Figure 4, but with an additional table detailing the number of subjects at risk at each follow up time point.
